# Isotactic Polybutene-1/Bamboo Powder Composites with Excellent Properties at Initial Stage of Molding

**DOI:** 10.3390/polym11121981

**Published:** 2019-12-02

**Authors:** Bo Wang, Fu-hua Lin, Yu-ying Zhao, Xiang-yang Li, Yan-chao Liu, Jing-bo Li, Xiao-Jing Han, Si-xiao Liu, Xu-ran Ji, Jun Luo, Ying-hui Wei

**Affiliations:** 1 School of Chemical and Biological Technology, Taiyuan University of Science and Technology, Taiyuan 030021, China; 13546474299@163.com (B.W.);; 2 School of Materials Science and Engineering, Taiyuan University of Science and Technology, Taiyuan 030021, China; kelin0514@163.com; 3 Shanxi Provincial Institute of Chemical Industry, Taiyuan 030021, China; 17835611229@163.com; 4 Guangzhou Fibre Product Testing and Research Institute, Guangzhou 510220, China

**Keywords:** isotactic polybutene-1, bamboo powder, mechanical properties, crystallization properties

## Abstract

Isotactic polybutylene-1 (iPB) has lots of advantages and is best used as hot water pipe. However, to transform into stable crystal form I, the iPB needs as long as 7 days. In this process, the irreversible damage brings great difficulties to the use of the iPB. The method which convert it directly into crystal I has shortcomings such as being requiring complex operation and being expensive. In this study, an innovative idea was put forward, not paying attention to the crystal transformation of iPB but only focusing on reducing the time it can be applied. In this study, bamboo powder was modified by the silane coupling agent KH570 (KBP) to prepare iPB/KBP composite. The infiltration test and Fourier transform infrared (FTIR) analysis showed that the hydrophilicity of KBP is greatly reduced, which can greatly improve the compatibility of the iPB and KBP. The tensile strength, tensile modulus, flexural strength, and flexural modulus of the composites storage for 3 days is equal to the pure iPB with storage 7 days with the KBP additions of 3%, 3%, 7%, and 5%, respectively. The heat deformation temperature (HDT) of the composite with 3% KBP after 1-day storage reached the value of pure iPB storage for 7 days. This provides more space and possibilities for the industrialization of the iPB. The crystallization behavior of iPB/KBP composites proves that the addition of KBP accelerates the crystallization rate of iPB, but the crystallinity of the iPB/KBP composites is not changed. The SEM photograph of iPB/KBP composites showed that when the KBP addition was low the compatibility between KBP and iPB was good. When the KBP addition was increased the agglomeration of KBP in the iPB was very obvious, which leads to the poor mechanical properties of the composite.

## 1. Introduction

Isotactic polybutylene-1 (iPB) is a kind of polybutylene-1 polymer with molecular chain regularity greater than 90% [1]. iPB has lots of advantages such as excellent mechanical properties, creep resistance, and stress-cracking resistance [2,3]. Especially, it can be used for a long time at 80–150 °C without deformation and can be completely recoverable [4]. Therefore, the use of iPB as a hot water pipe has incomparable advantages over traditional polypropylene. However, the iPB is a polymorphism polymer, which first forms thermodynamically unstable crystal form II (Tetragonal) after molding, which has disadvantages of low melting point and poor mechanical properties and then gradually transforms into stable crystal form I (Hexagonal) after 7 days at room temperature [5,6,7]. In this process, the irreversible damage of the iPB caused by external forces will bring great difficulties to the transportation and use of the iPB.

At present, researchers have been oriented toward finding solutions to improve the defect of the slow crystalline transformation of the iPB. Li et al. [8] studied the influence of iPB/iPP blend on the polymorphism of iPB under processing-relevant conditions. The form I can be produced directly by quenching mixed iPB/iPP melt down to temperatures below the upper critical solution temperature (The polymer blend system is completely compatible beyond this temperature) and the melting point, and the quenching temperature or quenching time has a great influence on the crystalline transformation of the iPB. Memory ordered melt (MOM) effect in polymer crystallization may be an effective approach to demonstrate whether the diffusive layer with intermediate order exists between crystal and melt or not. In this approach, the existence of ordered structure that cannot be detected directly is manifested through induced crystallization after temperature is lowered down. Su et al. [9] study the crystallization behavior of the iPB by used quench method. The result showed that after being partially melted at high temperature, a small portion of form I crystal of iPB-1 recovers back when the temperature is lowered. This is different from a disordered melt, which crystallizes into form II directly. Li et al. [10] were found that the iPB melt crystallization at 8 MPa CO_2_ and slow cooling rate can impel the crystalline transformation of the iPB to form I. Zhang et al. [11] used halloysite nanotube (HNT) and palygorskite (PGS) for nanofiller to reduce the crystalline transformation time of iPB. The results show that the PGS accelerates the transformation from crystal form II to I for PB-1 more efficiently than HNT.

Although the researchers have provided a series of methods to promote the crystalline transformation of the iPB, there are some shortcomings, such as complex operation, high cost, and so on. Since the crystalline transformation of the iPB is so difficult, can we change our thinking? Which kind of reinforcement filler can be selected to prepare iPB composite? It can not only ensure that the advantages of its complete recovery are not affected but also that it can be used for a short time after forming. In this way, the excellent performance of the iPB can be maximized and the industrial production cannot be affected. If this goal can be achieved, then the shortcomings of the slow crystalline transition of the iPB are less important.

As a kind of biomass material, bamboo has lots of advantages such as abundance, low density, low cost, biodegradability, and excellent mechanical properties [12]. In recent years, bamboo powder (BP) was widely used for filler of the polymers which can entrust better mechanical properties, crystallinity properties, and biodegradability properties of polymers [13,14,15]. Based on the superior properties of bamboo powder and its successful application in polymers, it is expected that BP will greatly improve the mechanical properties of the iPB at the initial stage of molding which can be used with a short waiting period.

As we know, BP is mainly composed of cellulose, hemicellulose, and lignin which have strong polarity. Inevitably, the interfacial compatibility between polarity BP and non-polarity iPB was poor [16,17,18]. The good mechanical properties of the composite may be invalid under the poor interfacial compatibility. Furthermore, it may accelerate the fracture of the composite when the composite is subjected to the external force. Silane coupling agent is a kind of compatibilizer that was widely used in inorganic materials/polymers composites [19,20,21] and nature Fiber/polymers composites [22,23,24,25]. In terms of mechanism, silane coupling agents react with hydroxyl groups on the molecular chain of natural fibers, and the composites with better compatibility are obtained [26].

In this study, the BP was modified by silane coupling agents KH570 to prepare KBP, and the iPB/BP composite was prepared by twin screw extruder. The performance change of BP modified by KH570 was characterized by Fourier-transform infrared spectroscopy (FTIR) and water contact angle measurement. The effect of KBP addition on the mechanical properties was investigated in detail. Moreover, the crystallization behavior and the compatibility of the iPB/KBP composites were discussed.

## 2. Materials and Methods

### 2.1. Materials

iPB (P5050) was purchased from Mitsui Chemical Inc. γ-(Methacryloxypropyl) trimethoxy Silane (KH570) was purchased from Nanjing Xuyang Chemical Co., Ltd. (Nanjing, China). Ethanol was purchased from Tianjin Damao Chemical Reagent Factory (Tianjin, China). The bamboo powder (BP) was utilized the residual from moso bamboo manufacturing.

### 2.2. Preparation of KBP

First, the bamboo powder (BP) was washed and dried to remove the dust, then crushed in a ball mill for 24 h (DECO-PBM-V-0.4L, Changsha Deco Equipment Co., Ltd., Changsha, China). Then the powder is sifted and selected the particle size of the powder is less than 200 mesh (BP). Second, The KH570 was dissolved in an ethanol solution to prepare KH570-ethanol solution with 10% mass fraction. At last, the BP and KH570-ethanol solution with a mass ratio of 100:1 (*w*/*w*) were mixing modification in the high-speed mixer using spray method (SHR-5A, Laizhou Shenlong Chemical Machinery Co., Ltd. Laizhou, China) for 2000 rpm and 30 min. The KBP was obtained through the above process.

### 2.3. KBP Characterization

#### 2.3.1. Infiltration Test 

The BP and KBP samples were press into a sheet (1 mm) using a tablet press (769YP-10T, Shanghai Xinnuo Instrument Equipment Co., Ltd., Shanghai, China). The infiltration test was measured by the sessile drop method with water as the solvent at ambient temperature using a water contact angle measuring instrument (DSA25, Kruss, Germany).

#### 2.3.2. FTIR Analysis

The FTIR of the BP and KBP was performed on a FTIR spectrometer with a diamond ATR accessory (Nicolet iS50, Thermo Scientific Inc., Norwood, MA, USA) using 32 scans per sample.

### 2.4. Preparation of iPB/KBP Composites

The iPB and KBP proportionally added to twin-screw extruder (TSH25, Nanjing Chuangbo Machinery Equipment Co., Ltd., Nanjing, China) for extrusion, strip casting, granulation and drying, the dry pellets (iPB/KBP composites) were obtained. For the extrusion process, the temperature for each division of the twin-screw extruder set as 110, 140, 160, 170, 170, and 160 °C, respectively. The formula of the iPB/KBP composites was shown in Table 1. Then the pellets were molded for standard test specimens by an injection machine (MA1200II, Haitian Plastic Machinery Co., Ltd., Ningbo, China) with an injection pressure of 12 MPa at 150 °C.

### 2.5. Mechanical Properties of iPB/KBP Composites

The universal testing machine (M10, Instron Company, USA) was used to evaluate the tensile properties and flexural properties of the iPB/MCC composites at 25 °C according to GB/T 1040-2006 and GB/T 9341-2008. The crosshead speed was set as 10 mm/min. The impact strength of the composites was measured by an impact tester (Ceast 9050, Instron Company, Waltham, MA, USA) with an 11-J capacity at a maximum pendulum height (150°) at room temperature according to GB/T 1043.1-2008.

The mechanical properties of iPB/KBP composites were tested at different storage times after molding (0, 1, 3, 5, 7 days). Ten samples for each iPB/KBP composites were measured and the average values were recorded.

### 2.6. Thermal Property of the iPB/KBP Composites

Thermal property is an important index of iPB as an application of hot water pipes. The Heat distortion temperature (HDT) of the iPB/KBP composites was measured by HDT/VICAT equipment (HV500, CEAST, Italy) according to China National Standard-Plastics-Determination of temperature of deflection under load (GB/T 1634.2-2004). The HDT of iPB/KBP composites were tested at different storage times after molding (0, 1, 3, 5, 7 days). Ten samples for each iPB/KBP composites were measured and the average values were recorded.

### 2.7. Crystallization Behavior of the iPB/KBP Composites

The crystallization behavior of the iPB/KBP composites was performed on a DSC (DSC1, Mettler Toledo Instrument Inc., Zurich, Switzerland). The iPB/KBP composites were heated to 180 °C at 30 °C/min, maintained for 3 min in order to erase the thermal history. Then, the sample was cooled to 30 °C at 10 °C/min. The whole process was under nitrogen flow (50 mL/min). The curve of cooling process (crystallization process) was recorded for subsequent data analysis.

### 2.8. Scanning Electron Microscopy of the iPB/KBP Composites

The distributed state of KBP in iPB was observed by scanning electron microscopy (SEM) (S-4800, Hitachi, Tokyo, Japan) at an accelerating voltage of 10 kV. 

## 3. Results and Discussion

### 3.1. Infiltration Test of BP and KBP

**Figure 1:**
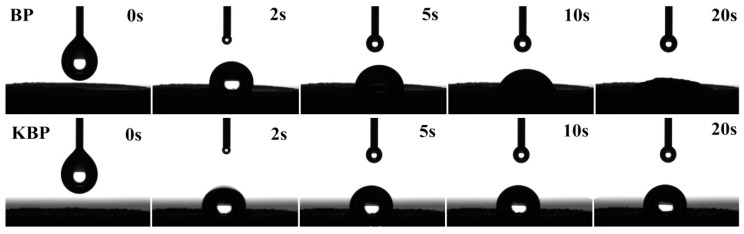
Infiltration test of BP and KBP.

The Infiltration test is often used to evaluate the hydrophobicity/hydrophilicity of fibers. Figure 1 presents the infiltration test of BP and KBP at different time. As we can see, the wettability by water of KBP is decreased compared with that of BP, indicating that the modification of KH570 improves effectively the hydrophobicity of BP. The decrease of wettability by water may be attributed to the fact that the introduction of KH570 can lead to the surface of BP changing from hydrophilic to hydrophobic because of the presence of organic functional groups of KH570. In summary, the organic functional groups in KH570 improve the hydrophobicity of BP evidently.

### 3.2. FTIR Spectra of BP and KBP

**Figure 2:**
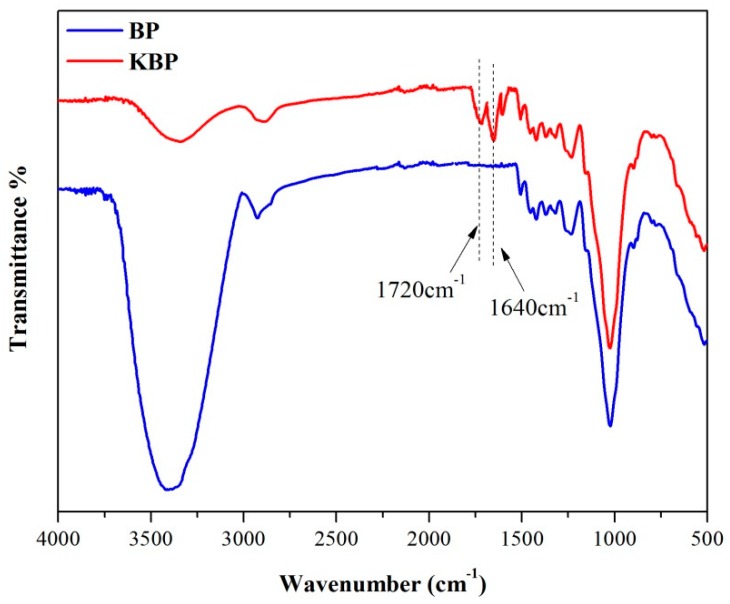
FTIR spectra of BP and KBP.

**Figure 3:**
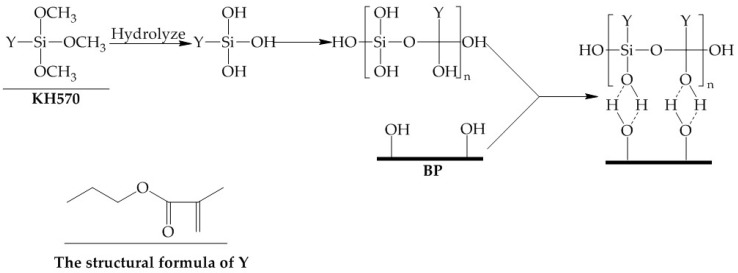
The reaction mechanism of BP and KH570.

The FTIR spectra (Figure 2) provided information on the change of BP chain structure groups before and after modification. As shown in Figure 2, the –OH stretching vibration at 3400 cm^−1^ can be found in the FTIR spectra of BP and KBP. More importantly, the absorption peaks of –OH decreased significantly after modification. Moreover, the new peak attributed to C=O and C=C stretching vibration has appeared at 1720 and 1640 cm^−1^, respectively [27]. The result indicated that the KH570 reacts with hydroxyl groups on the surface of BP. The hydrophilicity of KBP is greatly reduced. The reaction mechanism is shown in Figure 3 [28]. 

### 3.3. Mechanical Properties of iPB/KBP Composites

**Figure 4:**
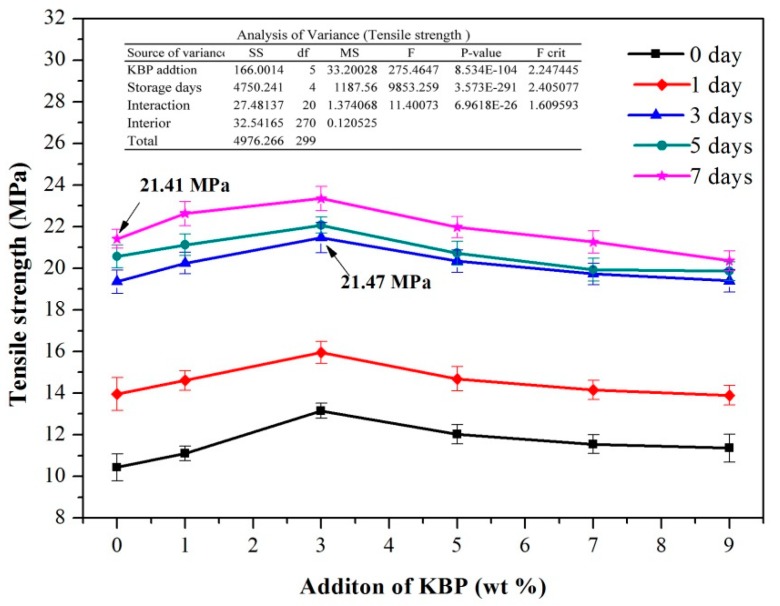
Tensile strength of the iPB/KBP composites

The tensile strength change of the iPB/KBP composites with the addition of KBP and the storage days is shown in Figure 4. It can be seen from Figure 4 that the tensile strength of the composites increases sharply at first and then slowly with the increase of the storage days. After 7 days, the tensile strength of pure iPB increased from 10.43 to 21.41 MPa (increased by 105.27%), the iPB/KBP1 increased from 11.10 to 22.63 MPa (increased by 103.87%), the iPB/KBP3 increased from 13.15 to 23.35 MPa (increased by 74.91%), the iPB/KBP5 increased from 12.02 to 21.97 MPa (increased by 82.79%), the iPB/KBP7 increased from 11.54 to 21.26 MPa (increased by 84.23%), and the iPB/KBP9 increased from 11.36 to 20.36 MPa (increased by 79.23%). This phenomenon also reflects the feature that the iPB needs to be storage for a long time before it can be used properly. 

Moreover, with the increase of KBP addition, the tensile strength of iPB/KBP composites first increased and then decreased. No matter how the storage days are changed, the tensile strength of the composites with 3% KBP addition shows the maximum value. Compared with the pure iPB with the same storage days, the date increased from 10.43 to 13.05 MPa (increased by 25.12%) after 0 days, the date increased from 13.95 to 15.95 MPa (increased by 14.34%) after 1 day, the date increased from 19.35 to 21.47 MPa (increased by 10.96%) after 3 days, the date increased from 20.57 to 22.07 MPa (increased by 7.29%) after 5 days, and the date increased from 21.41 to 23.35 MPa (increased by 4.39%) after 7 days. This phenomenon showed that KBP acts as the load-carrying part (the stress concentration point) and produces more crazing and shear zone in the iPB which can absorb energy to ensure the composite is not damaged when it subjected to external force. However, with the increasing amount of KBP, the agglomeration of KBP in the iPB becomes more and more serious which results in the decrease of the tensile strength of the composites [29]. Meanwhile, the variance analysis of the tensile strength data in Figure 4 shows that the P-value of KBP addition and storage days was much less than 0.05. The variance analysis data show that the KBP addition and storage days have a significant influence on the tensile strength of iPB.

Surprisingly, it should be noted that the tensile strength of the composites with 3% KBP addition and storage of 3 days (21.47 MPa) is equal to the pure iPB with storage of 7 days (21.41 MPa). This date showed that the tensile strength of the composite with 3% KBP addition can meet the demand without storage of 7 days, which greatly shortens the waiting period.

**Figure 5:**
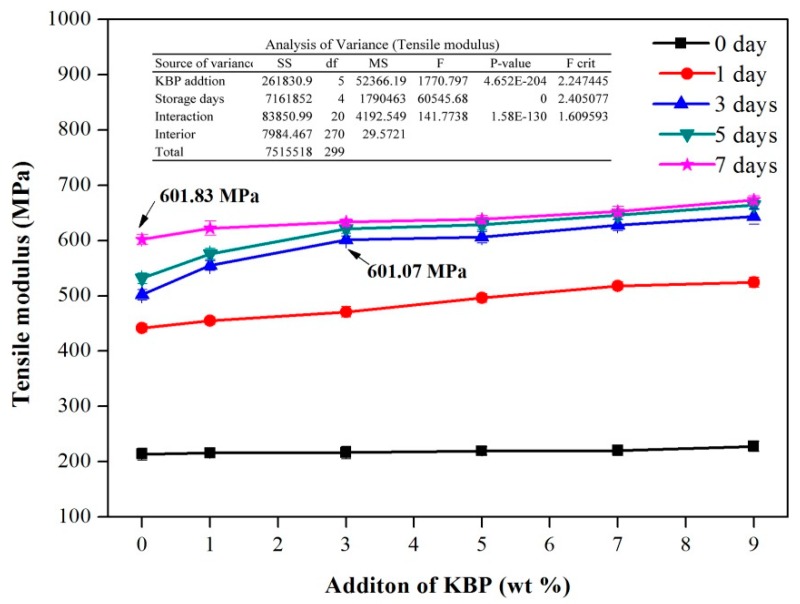
Tensile modulus of the iPB/KBP composites

The tensile modulus of the iPB/KBP composites with the addition of KBP and storage days is shown in Figure 5. Similar to the change of the tensile strength, the tensile modulus of the iPB/KBP composites increases rapidly and then slowly with the increase of the storage days. After 7 days, the tensile modulus of pure iPB increased from 213.69 to 601.83 MPa (increased by 181.64%), the iPB/KBP1 increased from 215.68 to 622.23 MPa (increased by 188.50%), the iPB/KBP3 increased from 216.44 to 633.53 MPa (increased by 192.70%), the iPB/KBP5 increased from 219.40 to 638.23 MPa (increased by 190.90%), the iPB/KBP7 increased from 220.40 to 652.67 MPa (increased by 196.13%), and the iPB/KBP9 increased from 227.65 to 673.25 MPa (increased by 195.73%).

Furthermore, with the increase of KBP content, the tensile modulus of iPB/KBP composites continues to increase. The tensile modulus of the composites with 9% KBP content reached the maximum value. Compared with the pure iPB with the same storage days, the date increased from 213.69 to 227.65 MPa (increased by 6.53%) after 0 days, the date increased from 441.84 to 524.60 MPa (increased by 18.73%) after 1 day, the date increased from 501.84 to 643.60 MPa (increased by 28.25%) after 3 days, the date increased from 531.78 to 664.41 MPa (increased by 24.94%) after 5 days, and the date increased from 601.83 to 673.25 MPa (increased by 11.87%) after 7 days. The main reason for this result is that the KBP as heterogeneous nucleation point in iPB matrix produces more microcrystals around the iPB matrix. Compared with the amorphous region, microcrystals have higher modulus which leads to the tensile modulus of composites increases continuously [30]. Meanwhile, the variance analysis of the tensile modulus data in Figure 5 shows that the P-value of KBP addition and storage days was much less than 0.05. The variance analysis data show that the KBP addition and storage days has a significant influence on the tensile modulus of iPB.

Similar to the tensile strength of the composites, the tensile modulus of the composites storage for 3 days was 601.07 MPa when the KBP content was 3%, while that of the pure iPB storage for 7 days was 601.83 MPa. This also shows that the tensile modulus of the composites can meet the needs of use without waiting for 7 days.

**Figure 6:**
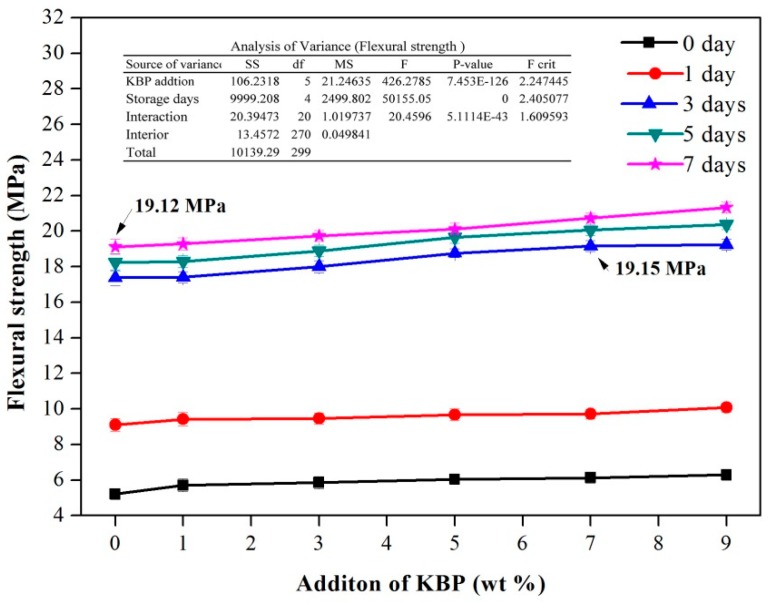
Flexural strength of the iPB/KBP composites

In the slow process of crystalline transformation, there is not only the possibility of fracture but also the problem of bending deformation. Therefore, the stability of the flexural properties of composites is also essential. Figure 6 shows the variation of the flexural strength of iPB/KBP composites with the increases of storage days. It was demonstrated that the flexural strength of the iPB/KBP composite increases with the increase of storage days. After 7 days, the flexural strength of pure iPB increased from 5.21 to 19.12 MPa (increased by 266.99%), the iPB/KBP1 increased from 5.70 to 19.28 MPa (increased by 238.25%), the iPB/KBP3 increased from 5.85 to 19.73 MPa (increased by 237.26%), the iPB/KBP5 increased from 6.04 to 20.12 MPa (increased by 233.11%), the iPB/KBP7 increased from 6.13 to 20.73 MPa (increased by 238.17%), and the iPB/KBP9 increased from 6.29 to 21.33 MPa (increased by 239.11%). The increase in flexural strength is much higher than that of tensile strength. From this point of view, the flexural resistance of the iPB/KBP composites will be greatly increased from the initial crystalline II to the final crystalline I, so that there will be no serious irreversible damage during transportation. 

Besides, the flexural strength of iPB/KBP composites increases with the increase of KBP content. The iPB/KBP9 obtained the maximum value of the flexural strength. Compared with the pure iPB with the same storage days, the date increased from 5.21 to 6.29 MPa (increased by 20.73%) after 0 days, the date increased from 9.10 to 10.09 MPa (increased by 10.88 %) after 1 day, the date increased from 17.37 to 19.23 MPa (increased by 10.71%) after 3 days, the date increased from 18.24 to 20.37 MPa (increased by 11.68%) after 5 days, and the date increased from 19.12 to 21.33 MPa (increased by 11.56%) after 7 days. This phenomenon also shows that the addition of KBP has a positive influence on the increase of the flexural strength of composites. Meanwhile, the variance analysis of the flexural strength data in Figure 6 shows that the P-value of KBP addition and storage days was much less than 0.05. The variance analysis data show that the KBP addition and storage days has a significant influence on the flexural strength of iPB.

Another important information from the figure can be apparent. The flexural strength of the composite with 7% KBP addition is almost the same as that of the pure iPB when it is placed for 3 days. This shows that the flexural strength of the composite can meet the requirements of use in 3 days and greatly improves the production efficiency in actual production. It provides conditions for the large-scale industrialization of the iPB.

**Figure 7:**
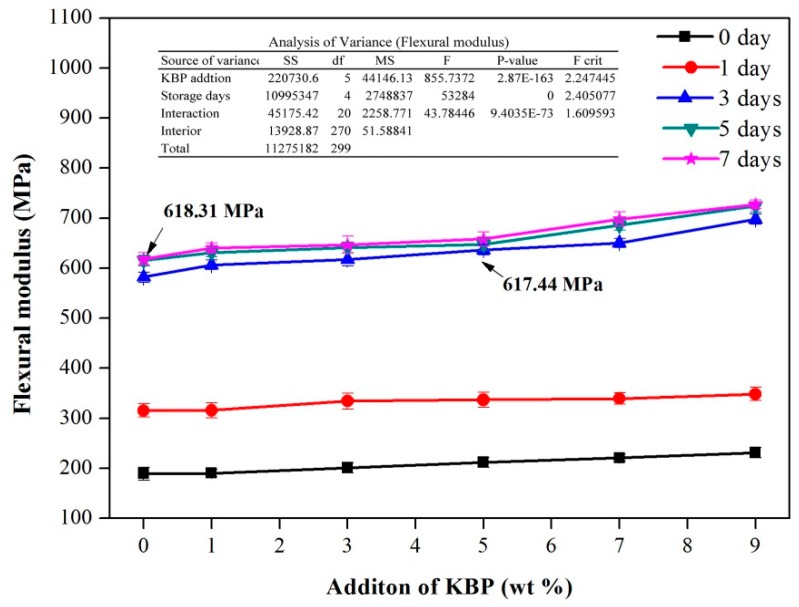
Flexural modulus of the iPB/KBP composites

The flexural properties of the material include not only the flexural strength but also the flexural modulus. The flexural modulus of the iPB/KBP composite is shown in Figure 7. It can be seen from the figure that the flexural modulus of the material increases with the increase of the storage days. 

After 7 days, the flexural modulus of pure iPB increased from 189.21 to 618.31 MPa (increased by 226.79%), the iPB/KBP1 increased from 190.15 to 640.31 MPa (increased by 236.74%), the iPB/KBP3 increased from 201.10 to 646.79 MPa (increased by 221.63%), the iPB/KBP5 increased from 211.89 to 658.12 MPa (increased by 210.60%), the iPB/KBP7 increased from 220.92 to 698.17 MPa (increased by 216.03%), and the iPB/KBP9 increased from 213.24 to 727.29 MPa (increased by 214.52%).

The flexural modulus of iPB/KBP composites continued to increase with the increase of KBP content. The iPB/KBP9 reached the maximum value of the flexural modulus. Compared with the pure iPB with the same storage days, the date increased from 189.21 to 231.24 MPa (increased by 22.21%) after 0 days, the date increased from 315.35 to 348.40 MPa (increased by 10.48 %) after 1 day, the date increased from 581.96 to 697.68 MPa (increased by 19.88%) after 3 days, the date increased from 614.96 to 723.88 MPa (increased by 17.71%) after 5 days, and the date increased from 19.12 to 21.33 MPa (increased by 17.63%) after 7 days. The crystal shape of iPB is spherulite with larger size. When KBP is added, the crystal cannot bypass KBP growth during the growth of iPB crystal. This makes the large spherulites of iPB become smaller spherulites or microcrystals. As the crystalline grain refines, the crystal of iPB transitions from the micron to the nanometer. The appearance of microcrystals also plays a self-reinforcing effect on the composites, which shows the increase of bending modulus. In addition, the variance analysis of the flexural modulus data in Figure 7 shows that the P-value of KBP addition and storage days was much less than 0.05. The variance analysis data show that the KBP addition and storage days has a significant influence on the flexural modulus of iPB.

Meanwhile, it can be observed in the figure that the flexural modulus of the pure iPB after 7 days reached 618.31 MPa, and the flexural modulus of the composite with a KBP addition of 5% is 617.44 MPa after 5 days. This value has been practically the same as the value of pure iPB storage for 7 days. This shows that the flexural modulus of the iPB/KBP composites storage for 5 days is also sufficient for practical applications.

**Figure 8:**
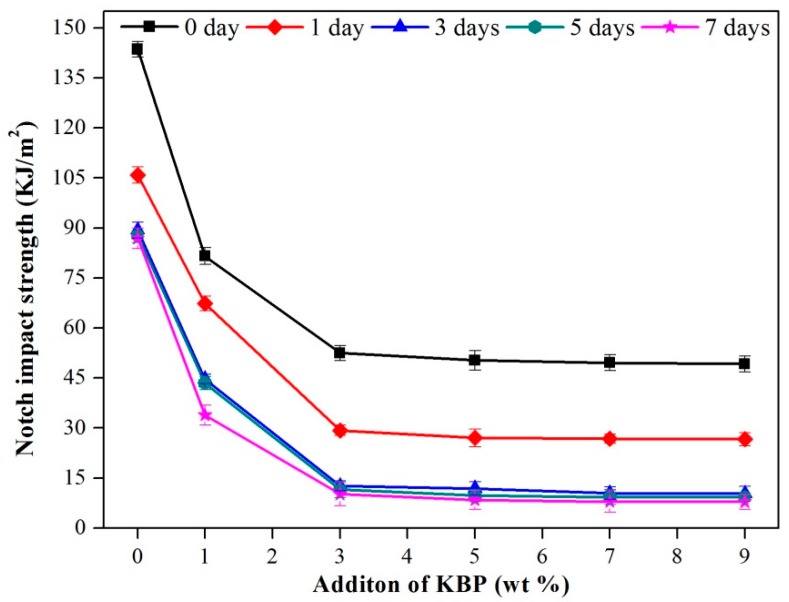
Impact strength of the iPB/KBP composites

The impact strength of the composites (Figure 8) showed a downward trend regardless of the storage days and the increase of KBP addition. With the increase of the storage days, the impact strength of the composites decreases, and as the amount of KBP increases, it first drops sharply and then tends to be stable. The reason for this phenomenon is that the addition of KBP promotes the crystallization of iPB, which makes the number of ordered segments of iPB molecules increase, and the amorphous portion capable of absorbing impact is less. When subjected to an external force, cracks are easier to appear, which leads to a reduction in impact strength [3]. In our previous research, the impact strength of PP (S1003) which was used for pipe is about 2.9 KJ/m^2^ [31] and the minimum value of the iPB/KBP composites is 9.219 KJ/m^2^. These data were shown that although the impact strength of the iPB/KBP composites decreases, it is still higher than the impact strength of the commonly used PP pipes, which can fully meet the daily use.

In brief, the storage process of iPB is the process from crystal II to crystal I. As mentioned in the foreword, crystal form II has the characteristics of poor tensile, flexural, and thermal properties. With the increase of storage time, the proportion of crystal form I in iPB matrix is increasing. As a result, the mechanical properties (excluding impact properties) and thermal properties of the iPB improve. This also proves that the rigidity of iPB crystal form I is much stronger than that of crystal form II. When KBP was added, it was inferred that the addition of KBP can accelerate the crystal transformation rate of iPB.

### 3.4. HDT of iPB/KBP Composites

**Figure 9:**
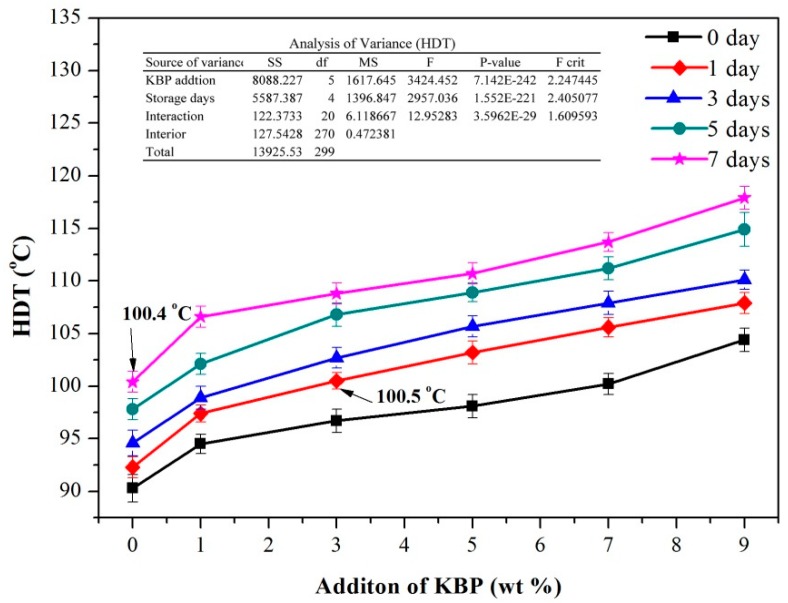
HDT of iPB/KBP composites.

As we all know, the iPB is used primarily in the field of hot water pipes in practical applications. Therefore, the thermal properties, especially the heat deformation temperature (HDT) property, are an important index for investigation. High HDT can provide a strong guarantee for its industrial development. The HDT of the iPB/KBP composite is shown in Figure 9. It can be observed in the figure that with the increase of storage days, the HDT of composites shows an increasing trend. 

After 7 days of storage, the HDT of pure iPB increased from 90.3 to 100.4 °C (increased by 11.18%), the iPB/KBP1 increased from 94.5 to 106.6 °C (increased by 12.80%), the iPB/KBP3 increased from 96.7 to 108.8 °C (increased by 12.51%), the iPB/KBP5 increased from 98.1 to 110.7 °C (increased by 12.84%), the iPB/KBP7 increased from 100.2 to 113.7 °C (increased by 13.47%), and the iPB/KBP9 increased from 104.4 to 117.9 °C (increased by 12.93%). This result fully demonstrates that the iPB must be placed for 7 days before it can reach a higher temperature to be more useful. In the process of iPB storage, the crystal form of iPB changes form II to I, and the most direct change is the increase of melting point. The melting point of crystal form I is about 120–135 °C, and that of crystal form II is about 110–120 °C [32]. It also shows that the HDT of iPB will increase with the increase of storage time.

Besides, with the increase of KBP content, the HDT of iPB/KBP composites also increases. When the addition of KBP reached 9%, the HDT of iPB/KBP composite reached the maximum. Compared with the pure iPB with the same storage days, the date increased from 90.3 to 104.4 °C (increased by 15.61%) after 0 days, the date increased from 92.3 to 107.9 °C (increased by 16.90 %) after 1 day, the date increased from 94.6 to 110.1 °C (increased by 16.38%) after 3 days, the date increased from 97.8 to 114.9 °C (increased by 17.48%) after 5 days, and the date increased from 100.4 to 117.9 °C (increased by 17.43%) after 7 days. The maximum HDT of the composites is 17.5 °C higher than that of pure iPB. This shows that the addition of KBP greatly promotes the HDT of the iPB. The reason for this phenomenon may be that the addition of KBP restricts the movement of the molecular chains of the iPB. In addition, the variance analysis of the HDT data in Figure 8 shows that the P-value of KBP addition and storage days was much less than 0.05. The variance analysis data show that the KBP addition and storage days have a significant influence on the HDT of iPB.

It is worth mentioning that the HDT of the composite with 3% KBP content reached 100.5 °C after 1 day storage and reached the value of pure iPB storage for 7 days. These data show that the iPB/KBP composite can be used in practice already after one day. This provides more space and possibilities for the industrialization of the iPB.

### 3.5. Crystallization Behavior of iPB/KBP Composites

**Figure 10:**
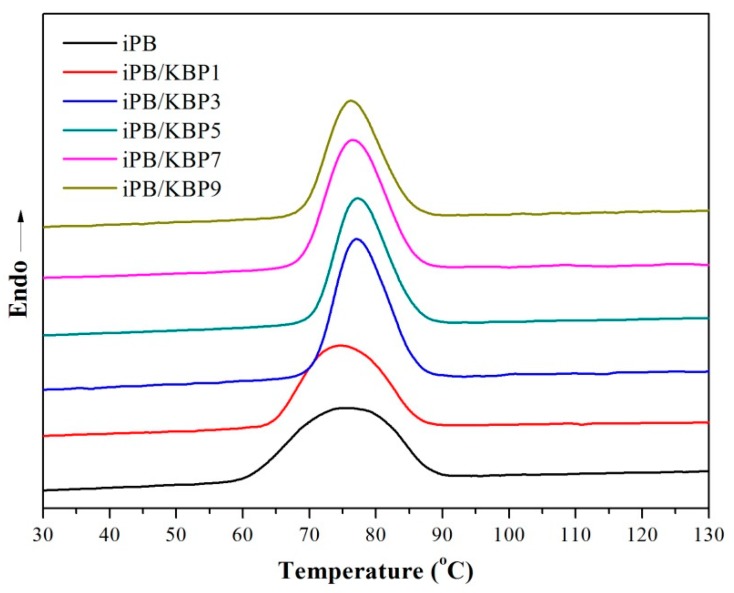
The DSC crystallization curves of iPB/KBP composites.

The crystallization curves and the crystallization peak parameters of iPB/KBP composites were shown in Figure 10 and Table 2. It can be seen from Figure 10 and Table 2 that the crystallization peak became narrow with the increase of the KBP addition when the KBP addition is less than 3%. Meanwhile, with the increase of the KBP addition, the crystallization peak basically remains the same. This phenomenon showed that KBP can shorten the crystallization time of iPB. Moreover, the crystallization end temperature (*T*_end_) and the crystallization peak temperature (*T*_p_) showed a trend to increase first and then decrease. The increases of *T*_end_ and *T*_p_ showed that the iPB/KBP composites have strong crystallization ability at higher temperatures. These phenomena all prove that the addition of KBP plays a very important role in accelerating the crystallization rate of iPB. However, the enthalpy of crystallization (Δ*H*) of the iPB/KBP composites is almost constant with the addition of KBP, which indicates that the crystallinity of the iPB/KBP composites not changed. The result just shows that KBP has little effect on the crystallinity of iPB. It can be seen from the DSC curves that the peak areas of each curve are basically the same, which proves that the crystallinity is basically the same. The narrow peak width indicates that the shorter the time to reach the same crystallinity, the faster the crystallization rate.

### 3.6. SEM of iPB/KBP Composites

**Figure 11:**
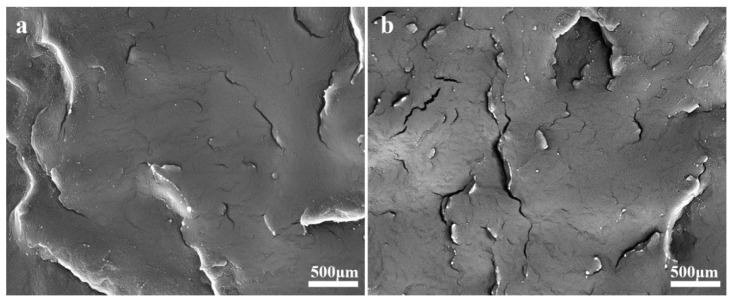
The SEM photograph of iPB/KBP composites (**a**) iPB/KBP3, (**b**) iPB/KBP9.

The SEM photograph of iPB/KBP composites was shown in Figure 10. It can be seen from the figure that the composite divided into white KBP phase and black iPB matrix phase in SEM photos. When the KBP addition was lower (Figure 11a) the KBP dispersed evenly in iPB. Moreover, the surface of the sample is smooth with fewer cracks, and KBP particles were smaller. This shows that the compatibility of KBP and iPB is good, and there is no KBP agglomeration in the composites. This enables KBP to enhance the iPB to obtain composite with excellent mechanical properties. But when the KBP addition was increased (Figure 11b), the agglomeration of KBP in the iPB matrix was very obvious, and the particle size of KBP kept increasing, and the surface of the sample was rougher with more and bigger cracks, which led to the poor interface compatibility between KBP and iPB, thus affecting the mechanical properties of the composite.

## 4. Conclusions

In this study, we change the existing research ideas of direct crystalline transformation of iPB and put forward that it can be used in the initial stage of molding (about 3 days) without considering the problem of crystalline transformation. The bamboo powder was modified by KH570 (KBP) to prepare iPB/KBP composite. The infiltration test and FTIR analysis showed that the hydrophilicity of KBP is greatly reduced. The compatibility of the iPB and KBP was greatly improved. The tensile strength, tensile modulus, flexural strength, and flexural modulus of the composites′ storage for 3 days is equal to the pure iPB with storage 7 days with the KBP additions of 3%, 3%, 7%, and 5%, respectively. The heat deformation temperature (HDT) of the composite with 3% KBP after 1-day storage reached the value of pure iPB storage for 7 days. This provides more space and possibilities for the industrialization of the iPB. The crystallization behavior of iPB/KBP composites proves that the addition of KBP accelerates the crystallization rate of iPB, but the crystallinity of the iPB/KBP composites did not change. The SEM photograph of iPB/KBP composites showed that when the KBP addition was low, the compatibility between KBP and iPB was good. When the KBP addition was increased the agglomeration of KBP in the iPB was very obvious, which led to the poor mechanical properties of the composite.

## Figures and Tables

**Table 1:** Formula of the iPB/KBP composites.

Sample	iPB (g)	KBP (g)
iPB	1000	-
iPB/KBP1	1000	10
iPB/KBP3	1000	30
iPB/KBP5	1000	50
iPB/KBP7	1000	70
iPB/KBP9	1000	90

**Table 2:** The crystallization peak parameters of the iPB/KBP composites.

Sample	*T*_end_ (°C)	*T*_p_ (°C)	Δ*H* (J/g)
iPB	58.1	75.1	33.1
iPB/KBP1	62.3	75.1	33.2
iPB/KBP3	67.6	77.2	33.3
iPB/KBP5	66.8	77.1	33.2
iPB/KBP7	66.4	76.4	33.1
iPB/KBP9	66.4	76.2	33.2

## Fundings

This research was funded by the financial support of the Scientific and Technological Innovation Programs of Higher Education Institutions in Shanxi (201802087), the Key Technologies R&D Program of Shanxi (201803D121044).
